# Suggestions and Kind Words from Friends

**Published:** 1872-07

**Authors:** 


### SUGGESTIONS AND KIND WORDS FROM FRIENDS.

"I hope you, gentlemen, will not consume much time or space in discussing taste with the editor of the Louisville & Richmond Medical Journal. You have as much right to your taste as he has to his. The profession of the South understand the cause of the opposition to the Companion and its editors. Please send me several copies of the Report of your troubles prepared by the Medical Society in your town."

"I see that the character of the senior editor has been assailed. Now, gentlemen, such assaults amount to nothing, save to "spot" the men who resort to such warfare. Wherever the "controversy" in regard to the college in your city is known, you are sustained, and your character remains untarnished. Character consists in the elments of the man, as displayed by what he does and the acts he performs. You have done but your duty, and the efforts to spatter you, in order to weaken your influence, and to cover the wrong, have all tended to intensify the truth. You have been persecuted, but you come out of it purified and justified before the profession."

"Dr. Gaillard's Review of the Atlanta pamphlet called, 'A Statement of Facts' is really amusing to those who are acquainted with the facts. I would suggest that you send the doctor a copy of the History of the Controversy in Georgia, prepared from the record."

"I am much pleased with your journal—its practical character and its devotion in culling the therapeutical truths emenating from all quarters. Your advertising department I find of great advantage, and one which I read with interest. I trust my brethren will avail themselves of the advantages it will certainly afford them—as almost everything can be found in it which they need for the successful prosecution of their profession. By restricting your advertising space to houses of undoubted reputation, you can and will be of great service to the physician—for which all should thank you. We should all endeavor to make it of mutual advantage to you in your efforts to serve the cause, and the gentlemen who call upon us for patronage through your journal."

"I, for one, thank you for your noble stand for the ethics. You have written many things which cannot fail to do good. I think, however, there is one subject you have not sufficiently enlarged upon, and one the profession do not generally regard with sufficient care. It is our duty to guard well and securely the *gates* of our profession. In doing this, we guard its honor. Call upon the profession, in thunder tones—and *repeat it!* REPEAT IT!!—never, under any stress or pressure whatever, to allow any young man to become a student in their offices, unless well and fully qualified by education and morals. And never—no never—allow any student to take even his first course of instruction in any but the best and most thorough colleges in the country. Let the profession make a compact—for the enobling of the cause, and for the blessings of science to the people—to do all in their power to cure the evils with which inferior schools are flooding the country.

Another thing: It is considered unprofessional for a physician to recognize a non-graduate, or any person who may have been guilty of irregularities in his professional conduct. The ethics holds us responsible for such affiliations. This is right. But why is it that some of the best colleges in the country receive and graduate students who have attended courses of lectures in schools all know to be undeserving and under the ban of the profession? Should colleges not act as individuals, and ignore the claims of inferior schools? If they will not take a high stand, ought not the profession, in a body, to direct their students to those colleges which do? Let the profession speak out—and not only speak, but act."

"If Dr. Gaillard endorses the course pursued by the Faculty of the Atlanta Medical College, it should be known, that gentlemen of other States who are in similar controversies may know what to expect from 'the largest medical journal in America.'"

"I advised you, some time ago, as a matter of information to the profession, that you publish the memorial upon which was predicated the action of the Medical Association in their dealings with the old Faculty of the Atlanta Medical College. This you declined to do for reasons which I now think were wise. My object was to have the profession fully advised, and the justice of the quarrel firmly established in the minds of medical men. Could they read it—although you will do best not to let it appear in your journal—they would see that the medical men of the State could not, in honor, escape the controversy without stultification, and that your course could not have been other than one of resistance to the abominable charges against you. A key to your opposition to it would have been furnished by the document—and your action fully vindicated."

"We have always believed you right in principle. Some of our physicians have been induced to believe, however, that you are too uncompromising even in the advocacy of truth—which has always sounded to me like saying a gentleman was too honest, or a woman too virtuous. Since Dr. Gaillard's attack upon your private character, they have changed their opinion, and are really anxious for you to publish the whole cause of the controversy. We think this is due the character of the profession of the whole State, as Dr. Gaillard's review of the statement of facts is a serious reflection upon the character of all who opposed the old Faculty of the Atlanta Medical College."

We thank our friend for the above information and suggestion. We are glad some of our mutual friends are willing to hear the whole truth; and if necessary they shall hear it. We have pitied them, and frequently laughed at their miserable policy. If we believe a thing to be right or wrong, we so declare it. We have never compromised the truth to make error respectable, or with the hope of securing permanent or even temprary peace. We are willing at all times to sacrifice our just claims and material interest for individual or general good, when it can be done without lending a part of the truth to error, that both may be equally respectable. That would be a compromise that could do no good to any one, and would necessarily make new issues and work to the injury of many. Peace can only be secured by strict adherence to the truth.

"Dr. Gaillard states that you, Dr. Powell, were expelled from the faculty of the college in your city (there is no college in this city now—it has been sold.—Eds.) How it was possible for Dr. G. to make this blunder—if a blunder—is beyond my comprehension. He has only to look up the facts embraced in the reports of Fulton County Medical Society, to be answered and the statement refuted. My advice is that you cease any further discussion with Dr. G. If he wishes to place himself wrong by endorsing the past actions of the old Faculty of the Atlanta Medical College, let him do it. He will see his folly ere long if he takes that direction."

This was handed to the Senior Editor by a friend, which makes it unnecessary for him to answer the charge, personally, as one of the editors of this journal.

"Ask Dr. Gaillard if any gentlemen connected with his College had been requested to resign his position because of his opposition to an amendment to the charter similar to the one obtained by the Faculty of the Atlanta Medical College, if it would not have brightened instead of stained his character? I do not desire this to be asked for the purpose of vindicating you and injuring Dr. Gaillard, but it is to learn if a rigid and uncompromising adherence to the fundamental principle upon which the safety of this government and the rights of its people rest—upon which the honor of the profession depends—and upon which the Christian religion alone can be maintained—is to stain a man in his estimation or the esteem of any true gentleman—all of which principles were ignored—destroyed—in the passage of the amendment, and the privileges it granted. A true answer to this question will perhaps satisfy him why so many honorable gentlemen have consented to become connected with "THE GEORGIA MEDICAL COMPANION, and why none are afraid to trust the interests of the profession and their honor in the hands of its Editors."

*Messrs. Editors:*

The Medical Profession of Georgia, and many in the Southern States, are amazed that the Trustees and Faculty of the Atlanta Medical College have permitted the building to be sold at sheriff's sale for the sum of $165 00. How is it that the State's property has thus been sacrificed? It may be that the late medical controversy may have destroyed the prospects of the college—hence the indifference of the Trustees and Faculty. Please inform your readers, if you can, what will be done in the premises. *.

It is a fact that the college building has been sold. We will state that we are informed that a majority of the Board have never fully understood the merits of the opposition of the profession to the College, and they will doubtless hold the Faculty responsible for permitting a great State interest to pass into private hands for a mere pittance.

*Dr. T. S. Powell:*

I regret to see that Dr. Gaillard, of Louisville, has succeeded in dragging you into a controversy about certain charges of a *personal nature*, which even your enemies have long admitted to be false. Had Dr. G. taken the trouble to read the published action of Fulton County Medical Society relative to these very charges, he would scarcely have had the temerity to attempt their resuscitation. That action was the result of close investigation, mature deliberation, and unbiased judgment, coupled with a thorough knowledge of all the facts in the case. The committee's report, upon which was based the Society's action, not only exhausted the argument, but was a most complete vindication of your professional integrity. It palsied the tongues of your traducers, and put a final quietus upon their fabrications. No man has yet attempted its refutation.

It is to be regretted that Dr. Gaillard, a gentleman whose intelligence and courtesy are seldom at fault, should seek to resurrect in Atlanta circles an issue so emphatically dead. You, Docto should have treated the effort with contempt. Why fight a battle already won? When you whipped it in Atlanta, you whipped it in Louisville, and you whipped it everywhere. Truth is ubiquitous. Yours, etc., *

The above is from the pen of one of the most accomplished writers and physicians of our State. We thank him for his kind expressions, and hope, with him, that no peaceful-minded gentleman will so far forget "good taste, decorum or propriety," as to attempt to re-open an old controversy, in which no credit can possibly accrue to any one personally, in such an effort, and which cannot fail to most thoroughly blemish the professional record of certain parties the "editor" of the "largest medical monthly in America" seems so anxious to justify and defend.

The above extracts and paragraphs are truly "suggestions and kind words from friends." For these our friends have our sincerest thanks. We publish them not as a compliment to these friends, or to compliment ourselves or journal, but with the same spirit and from the same motive with which we have warred against vice and error—that good may result to others. The Senior Editor could not have approved the manner in which the amendment to the charter of the Atlanta Medical College was obtained with less earnestness and energy, and have maintained his self-respect. He could not have claimed or deserved the confidence of the people, as a patriot or good citizen, had he endorsed it. He could not have claimed, upon the truest principles of honor and unsullied integrity, the respect or confidence of the humblest regular physician, and have endorsed the graduation of students under the broad and latitudinous powers and privileges of that amendment. He could never get his consent to conspire against the honor of his profession, and, with lifted arm, aid in its assassination. He could not have claimed to have been a civilized man and Christian gentleman while endorsing the more than heathen principle of pronouncing sentence of condemnation against his fellow-man without first meeting him face to face and affording the accused a fair and impartial hearing. If he has been wrong in all this—then truth, principle, law and Christianity are dilusions, vice a virtue, and honor a reproach.

